# Unlocking biomedical data sharing: A structured approach with digital twins and artificial intelligence (AI) for open health sciences

**DOI:** 10.1177/20552076241271769

**Published:** 2024-09-05

**Authors:** Claire Jean-Quartier, Sarah Stryeck, Alexander Thien, Burim Vrella, Jeremias Kleinschuster, Emil Spreitzer, Mojib Wali, Heimo Mueller, Andreas Holzinger, Fleur Jeanquartier

**Affiliations:** 1 Research Data Management, 27253 Graz University of Technology, Graz, Austria; 2 Institute for Medical Informatics, Statistics and Documentation, Medical University Graz, Austria; 3 130347 Research Center Pharmaceutical Engineering GmbH, Graz, Austria; 4 Institute of Technical Informatics, 27253 Graz University of Technology, Graz, Austria; 5 Division of Molecular Biology and Biochemistry, Medical University Graz, Austria; 6 Information Science and Machine Learning Group, Diagnostic and Research Center for Molecular Biomedicine, Medical University Graz, Austria; 7 Human-Centered AI Lab, Institute of Forest Engineering, University of Natural Resources and Life Sciences, Vienna, Austria; 8 Institute of Interactive Systems and Data Science, 27253 Graz University of Technology, Graz, Austria

**Keywords:** Artificial intelligence, human–computer interaction, sensitive data, usability, digital twin, cancer, disease, open science, metadata, reproducibility, FAIR

## Abstract

**Objective:**

Data sharing promotes the scientific progress. However, not all data can be shared freely due to privacy issues. This work is intended to foster FAIR sharing of sensitive data exemplary in the biomedical domain, via an integrated computational approach for utilizing and enriching individual datasets by scientists without coding experience.

**Methods:**

We present an in silico pipeline for openly sharing controlled materials by generating synthetic data. Additionally, it addresses the issue of inexperience to computational methods in a non-IT-affine domain by making use of a cyberinfrastructure that runs and enables sharing of computational notebooks without the need of local software installation. The use of a digital twin based on cancer datasets serves as exemplary use case for making biomedical data openly available. Quantitative and qualitative validation of model output as well as a study on user experience are conducted.

**Results:**

The metadata approach describes generalizable descriptors for computational models, and outlines how to profit from existing data resources for validating computational models. The use of a virtual lab book cooperatively developed using a cloud-based data management and analysis system functions as showcase enabling easy interaction between users. Qualitative testing revealed a necessity for comprehensive guidelines furthering acceptance by various users.

**Conclusion:**

The introduced framework presents an integrated approach for data generation and interpolating incomplete data, promoting Open Science through reproducibility of results and methods. The system can be expanded from the biomedical to any other domain while future studies integrating an enhanced graphical user interface could increase interdisciplinary applicability.

## Introduction

Data forms the basis for knowledge discovery and systems modeling.^1,2^ The open data movement has advanced scientific progress and has been accepted by some areas more than others.^3^ Sharing medical datasets facilitates the interconnection and combination of various disease-based information sources leading to new insights.^4^ In general, sharing digital artifacts supports the ongoing research process when overcoming the various concomitant challenges including technical, legal, and ethical issues.^5–7^ Privacy issues are particularly challenging in the health domain.^8–10^

This work describes an approach to sharing sensitive data by scientists also less confident about informatics facilitated by the use of a digital twin model for the exemplary application of generating synthesized biomedical data of human samples. It is intended to overcome the barrier of uncertainty about appropriate data processing for sharing in order to avoid a tendency not to share data at all. The following subsections will introduce the topics of general aspects of sharing digital research results, the necessity of high data quality, the importance of reproducibility, the human factor when it comes to using healthcare informatics systems, and the possibility of controlled data sharing as well as synthetic data.

### Past toward future sharing of research output

There are several efforts to novelize and create standards for reporting scientific research including underlying datasets and source code.^11–13^ Guidelines for reporting in different fields of health research define best practices for specific scientific domains.^14–16^ Such guidelines comprise aspects for displaying data and reporting data analysis including statistical methods, given another example in experimental biology.^17^ Additional suggestions have been reported for machine learning practitioners in computational biology and related sciences.^18^ Directives introduce guidelines to reporting specific items such as transparency of artificial intelligence (AI) models particularly in the setting of critical domains such as clinical applications.^19–21^ Minimum information for Medical AI Reporting comprising the essential components of AI solutions in healthcare have also been described.^22^ Thanks to popular recent successes (e.g. ChatGPT), AI is now well-known and accessible and it is obvious that integrating controlled health data for AI models poses unimagined new solution possibilities.^23^

In the advancement of digital twins for biomedical data sharing, the methodological aspects of AI play a crucial role, underpinned by cutting-edge AI technologies and sophisticated algorithms.^24^ At the core of these methodologies is the application of deep learning, particularly convolutional neural networks (CNNs) and recurrent neural networks (RNNs), which are adept at processing and interpreting complex, high-dimensional data inherent to biological systems.^25^ CNNs excel in identifying patterns and features within spatial data, making them ideal for medical imaging analysis, while RNNs are suited for temporal data analysis, crucial for understanding dynamic physiological processes over time. Furthermore, the emergence of generative adversarial networks has introduced novel possibilities for synthetic data generation and augmentation, enhancing the robustness and diversity of datasets for training digital twins. On the algorithmic front, advancements in reinforcement learning algorithms, such as deep Q-networks and policy gradient methods, have enabled the optimization of treatment strategies and decision-making processes in a clinical context. These AI methodologies are not static; continual improvements and innovations, such as attention mechanisms and transformer models, offer unprecedented accuracy and efficiency in data processing and analysis. Moreover, the integration of explainable AI (XAI) principles is gaining momentum, addressing the need for transparency and interpretability in AI-driven medical applications. Through techniques such as feature importance scoring and model-agnostic methods, XAI aims to make the decision-making processes of AI models more comprehensible to human experts, thereby enhancing trust and reliability in digital twin technologies which is now a legal requirement.^26^ Collectively, these methodological advancements in AI fundamentals are shaping a new frontier in personalized medicine, where digital twins serve as a nexus between cutting-edge AI technologies and the intricate realities of human health.

Universally, principles for findability, accessibility, interoperability, and re-usability (FAIR) have been specified for all digital artifacts accompanying scientific publications.^27,28^ General reporting of scientific findings and the steps that lead there comprising all digital artifacts can be accomplished and are supported by computational notebooks that are designed to support the interactive development and publication of computation details and its underlying scientific workflow.^29,30^

Making data available and reusable in different contexts to the scientific research community can be successful providing certain data exploration abilities of researchers while it depends on the quality of datasets. Data quality is an essential component described in several standards including the generic international standard of master data management, the ISO 8000 series,^31^ ISO 9000,^32^ as well as ISO 25000^33^ for System and Software Quality Requirements and Evaluation, and other more discipline-specific standards such as ISO 19157.^34^ Preprocessing of heterogeneous datasets is a critical step in every data analysis.^35,36^ This includes the aspect of completeness and clearing data from empty fields. Sufficient and well-prepared data is essential for accurate AI models or their validation.^35^

Scientific integrity can function practically by adhering to standards encompassing reproducibility next to objectivity, clarity, and utility.^37^ While FAIR repositories are one step toward reproducibility, specialized tools are necessary to ensure the portability of software and system dependencies for code execution.^38^ Model reproducibility and reuse by the international community is of specific interest in particular in the biomedical domain.^39^ Machine learning platforms have been proposed as a framework toward reproducibility out-of-the-box, still they cannot function as such without further trust-ensuring mechanisms.^40^ Computational notebooks thereby enable the underlying transparency^41^ while data platforms allow for a user-friendly and controlled integration and exchange of digital artifacts.^42^ Cyberinfrastructures provide those tools to scientists who do not have knowledge of command line computing.^43^

Human–computer interaction is important for an efficient usage of new developments, in particular, true for digital healthcare applications.^44^ Context to data is not only a matter of reproducibility, but also of usability.^45,46^ In order to make use of heterogeneous data, especially in the biomedical field various approaches including visualization features have been proposed.^47,48^ Virtual lab books, for example, via CyVerse offer ease-of-use for biomedical scientists and novices to data science, enable open collaboration to cooperative data analysis, transparent dissemination including supplementary material, enabling interaction between users, present documentation, and foster learning and understanding of the underlying methodology. Other virtual labs with controlled access supporting Jupyter exist, f.i. Google Colab.^49^ Since Colab requires a contract between Google and the respective user, it is not always appropriate for sensitive data.^50^ This study uses CyVerse Austria, a shared local platform for research data management.^51^ It holds the advantage of institutional storage in consideration of data privacy issues complying with national or, respectively, European legislation.

### Controlled data sharing and digital twins

Biomedical research often produces sensitive data.^52^ Human-related data involve ethical, legal, and social sensitivities, hindering researchers from sharing derivatives due to the complexity of processing.^53^ Though still sharing of biomedical data is essential for the development of novel treatment options, it is hindered by the civil view on data sharing for health research.^54^ Based on the degree of data use restrictions, different approaches to sharing data could overcome retention of data by its generator facilitating a secondary use.^55^ Various methods have been suggested to approach the use of sensitive data^56^ including federated data integration into graph-based systems.^57^ Controlled data-sharing initiatives such as the Personal Health Train tries to circumvent privacy issues through a FAIR distributed data analytics infrastructure while data remains with their owners.^58^ In general, federated biomedical models and multi-party computation approaches are still under development to overcome obstacles such as pending security issues.^59,60^

Furthermore, synthetic data has attracted attention in several disciplines and recently particularly for biomedical research.^61^ Algorithmically generated data can be used for testing, validation, and model training, also in case of limited dataset availability or in case of privacy issues.^62^

The idea behind digital twins has been introduced in the last century.^63^ Digital twins are a concept coined at the beginning of the 21st century that are starting to see widespread applications in many diverse fields that concern themselves with large amounts of data and/or complex systems. Thereby, applications of digital twins range from simulation to analysis and prediction.^64,66-68^ In general, the concept of digital twins refers to a virtual (digital) representation of a system or object, connected to a physical counterpart, using real-world data to simulate and analyse not only the actual state of that system or object, but also possible future states.^68^ In conjunction with state-of-the-art machine learning technologies and data analysis tools, digital twins allow the understanding and prediction of possible treatments for illnesses, the likely ramifications of a business decision or the training of operators and maintenance personnel for critical systems under controlled circumstances^68,69^ Currently, digital twins are often used for simulation purposes and especially in the engineering and heavy industry the term and its application have found their way into day-to-day work.^70^ Perhaps less expected but even the agricultural sector is starting to see the application of digital twins.^71^ Another field where many digital twin-related projects are being spearheaded is the medical sector.^72,73^ With concerns about patient confidentiality and highly personalized treatments many digital twin-related projects are being explored, such as cancer patient digital twins are yet to be created.^64,65^

This paper examines an application of digital twins in the field of biomedical research, with a focus on accessible AI for non-information technology (IT)-affine researchers and the potential to tackle sensitive data issues. Our introduced pipeline allows us to substitute various forms of empty fields and compare model performance and accuracy. It is presented with a use case of cancer research assessed through quantitative and qualitative measures for modeling performance as well as its human-centered design applicability to biomedical and health systems.

## Materials and methods

**Figure 1.**
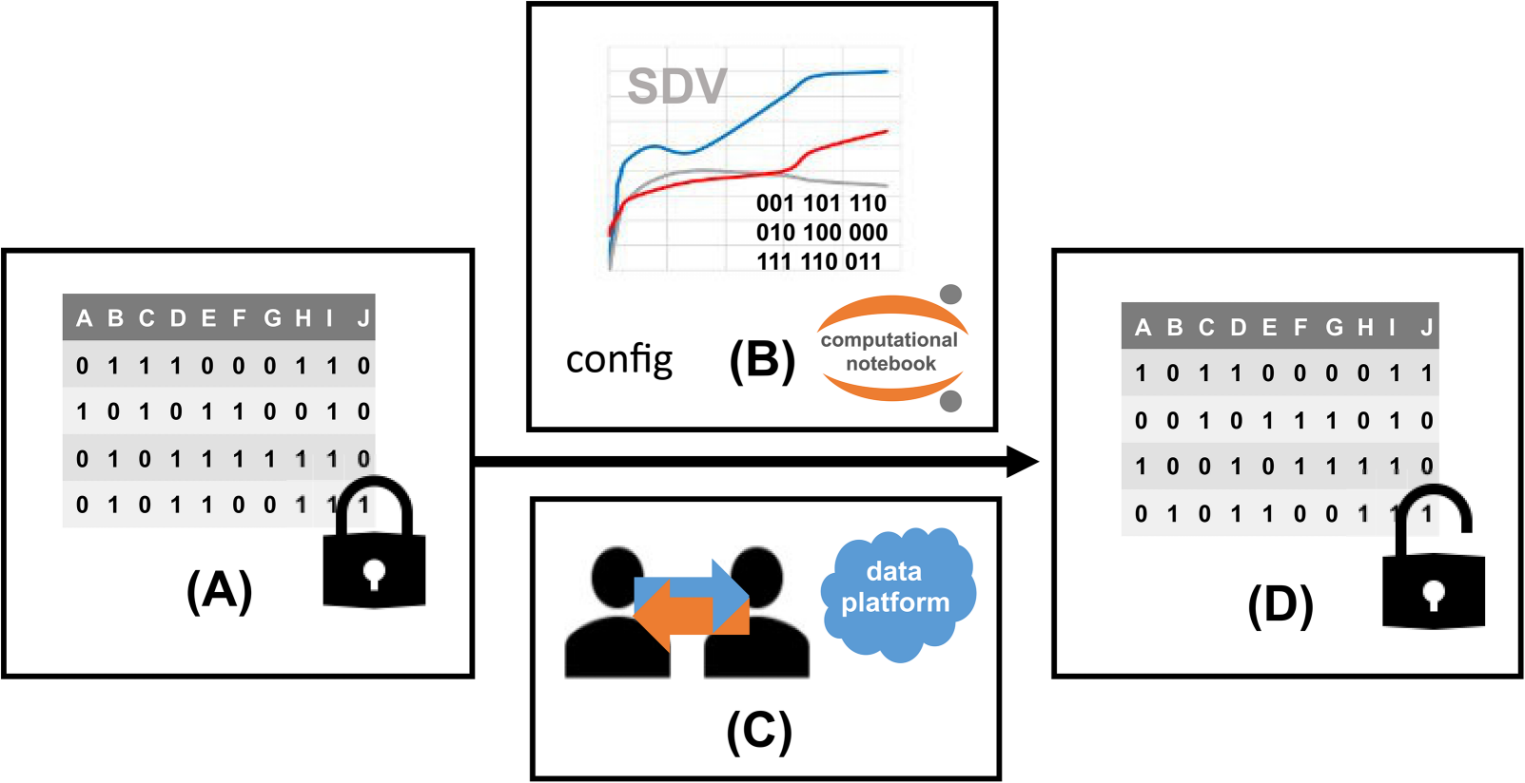
Overview of workflow for synthetic data generation based on computational notebook sharing: (A) controlled tabular data is used as input for (B) a digital twin model with features specified in a separate configuration file, (C) presented and shared as a container over a data analysis platform (D) generating synthetic data.

This article describes an approach to support sharing of controlled data via a user-friendly digital twin. The underlying pipeline is summarized in Figure 1. Methods along (A) data input, (B) model implementation, (C) sharing platform, and (D) output comparison are described beneath.

The framework for this pipeline was conceived and originally constructed in spring 2022 and continuously refined during user tests between 2022 and 2023 to implement improvements until the final refinements in April 2023.

### Data input

Various datasets containing tabular data of biochemical and clinical information on cranial cancer patients^74–78^ have been retrieved from cBioPortal.^79,80^ Next to “participant” and “study sample” identifiers there are columns that specify the exact type of tumor and the current clinical state of the patient in the form of the patients’ survival time since diagnosis as well as the status of alive or deceased and many more dataset-specific clinical information dependent on the study. For the classification, there are multiple columns which specify mutations, their count and type. These had been retrieved through the gene query of selected and top mutated genes: TP53, telomerase reverse transcriptase (TERT), isocitrate dehydrogenase 1 (IDH1), ATRX, PTEN, TTN, EGFR, MUC16, NF1, PIK3CA, CIC, RYR2, RB1, NOTCH1, PIK3R1, AHNAK2, AHNAK, LRP2, FLG2, OBSCN, and MUC12. These could then be downloaded from the download tab under the option of mutations as tab-delimited format. The file contains study and sample IDs next to gene status or specified mutations, which were integrated with the clinical information. In the case of tests for mixed and incomplete data, all studies have been combined. In the case of qualitative assessment of synthetic data from complete input data, only the dataset by the glass consortium^78^ has been utilized.

### Implementation

In order to generate synthetic data the synthetic data vault (SDV), a multivariate model allowing to deploy several types of datasets, has been chosen from a multitude of available models.^62,81^ The state-of-the-art tool for synthetic data generation that is available under MIT license https://sdv.dev/SDV/^95^ has been evaluated,^94^ and exemplary used and refined to apply to the selected target group.

The SDV framework constitutes a multivariate database modeling method that builds generative models for individual tables, and it additionally performs extra computations to account for the relationships between them, using a method called conditional parameter aggregation.^95^

The model is built by applying the Tabular Preset method from the Synthetic Data Vault Python (in v0.18.0) library to the given dataset. Modeling time is optimized through the method preset FAST_ML (Machine Learning). Further methods include model fitting (model.fit) and sampling (model.sample). After the training of the model, the creation of synthetic data is started by using the sample method.

To enable more specific data generation our metadata constraints are displayed in the form of a configuration file. This config file is a structured JSON file, which holds the following parametrization:

- Input file: Path + name of the input data file that shall be used for data generation, given as a string.
- Input column separator: Most csv files have a “,” separator, however, some also use semicolons “;” or tabs “” instead. Please then specify with this config parameter.
- Output file: Path + name of the file where we want the output to be written to.
- Percentage: A floating point number, which designates the percentage of non-NaN in input to be taken into account. The maximum percentage of non-numeric types that you want to allow in any column in input, as float, ranging from 0.0 to 1.0. entries a column must have to be considered for data generation.
- n_samples: The number of new lines of data the program should generate for you, given as a positive integer.
- Features: The headers of the columns of your input file you want to include in the new data as value pairs. Each feature is a key, where the value pair can be either “categorical” and then none type or a “numerical” value and then the appropriate data type (float—int).

#### Configuration

When starting the program, the config file is read, thus giving the program access to the location of the input and output file as well as a basic configuration that is used to generate data. This configuration constitutes itself via three parameters. The validity of the columns and sample number are designated by the “Features” parameter. The “Percentage” parameter of the configuration defines the number of entries in a given column to be a non-NAN value, described more in detail beneath under metadata.

#### Metadata

The metadata is then generated by the definition of the user in the config file with various configurable parameters. The parameter “Input File” defines the path and name of the input data file that shall be used for data generation, given as a string. Through the “Input Column Separator” the separator can be set, s.a. semicolon or tab with “∖t” instead of a comma for the default “,”. The parameter “Output file” defines the path and name of the file where the output will be written. “Percentage” sets a floating point number that designates the percentage of non-NAs in input to be taken into account. It depicts the maximum percentage of non-numeric types in any column of the input, as a float, and can be specified within the range from 0.0 to 1.0. to be considered for data generation. “The_samples” defines the number of new lines of data to be generated, given as a positive integer. In the section “Features” for every type, there is a subtype that defines the feature the user is interested in. The type distinction is made between “categorical” and “numerical.” The type numerical has also a subtype that defines the data type of the feature as “integer” or “float.”

#### Preprocessing

In the preprocessing step, the program only reads the chosen columns and these chosen columns eliminate invalid ones. The next preprocessing step the program has to perform is to remove the NAs and interpolate. The interpolation is done using the Python library Pandas with the “pad” setting, using existing values to fill in NaNs. After the interpolation, the validation set is created by using 30% of the given data and using the sample method of pandas.

#### Model

For the model, the “Tabular Preset method” of SDV is used. With the parameters “FAST_ML” and the metadata, a model is created to generate new synthetic data. The first parameter defines the type of the preset which is “fast machine learning” in this case and the second is the previously defined metadata. Then, data is fitted to the model and new synthetic data with the sample method is created. To preserve the data it is saved as .csv file. For the evaluation of the generated data, the method evaluated from the SDV library is used and metrics for the newly generated dataset are assessed by “KScomplement.”

### Data platform

CyVerse Austria^82^ (access at https://de.cyverse.tugraz.at, project source https://github.com/cyverse-at) runs inside Graz University of Technology intranet. Registered accounts can enter the system and receive shared data and analyses from other registered users. The notebook data_synthesizer.ipynb file together with the config.json , a dummy input—exemplary cancer dataset—named combined.csv , and a readme.md for initial instructions on how to use the model (and information on the requirements) are shared as analysis between users through the discovery environment. The latter depicts the workspace provided with a user interface of the cyberinfrastructure. The distributor guides the new users through the cyberinfrastructure to start the Jupyter application with the shared analysis. The notebook can be found in the data/input folder and is presented directly in the Jupyter workspace as a second tab next to the readme file. For writing permissions, which depends on the choice between read/write/own by the sharer of the container, the above files inside the container can also be copied to the new user’s home directory where their own data input can be integrated and new output files can be saved from the model.

### Assessment

The quantitative model assessment was performed by evaluation scores that have been determined using metrics and evaluated from the sdv packages, next to pandas, matplolib, numpy, and seaborn. Further details and versions are noted at https://github.com/dude2033/data_synthesizer.

Validation of the output was also performed qualitatively by inspecting and comparing results with observations in input data. Specific features describing input data characteristics were isolated from the literature and compared to in output data. Correlations between variables “TERT,” “IDH1,” “Diagnosis Age”/“Age at first diagnosis”/“Age Combined,” and “OS Months”/“Overall Survival (Months)” depend on variables of the various input datasets. Significant difference was tested using Mann–Whitney–Wilcoxon test two-sided from statannotations, with ns: p≥5.00e−02 , * :1.00e−02<p≤5.00e−02 , **: 1 .00e−03<p≤1.00e−02 , ***:1 .00e−04<p≤ 1.00e−03 $p\leq 1.00\times 10^{-3}$ , and **** :p≤1.00e−04 .

We studied feasibility and user experience (UX) by conducting a thinking aloud (TA) test^83^ with five participants, involving an UX expert, accompanied by a system usability scale (SUS) assessment,^84-86^ a de facto standard to quickly measure how people perceived the usability of a system^87^ with regard to user preference.^88^ TA tests took place online, on different days in March 2023 during working hours, utilizing video conferencing software with camera, microphone, as well as screen share functionality. Each TA test took about 45 min. Primary selection criteria for test users were based on some experience in data handling in the field of biomedical research. PhD students and postdocs have been specifically targeted for this purpose. Test participants consisted of three women and two men.

The test was planned, and moderated with the help of a UX expert, the last author, and pilot-tested with the first author.

The TA test plan can be found in the supplemental file .

## Results

### Comparison of synthetic data generation through model optimization

**Figure 2.**
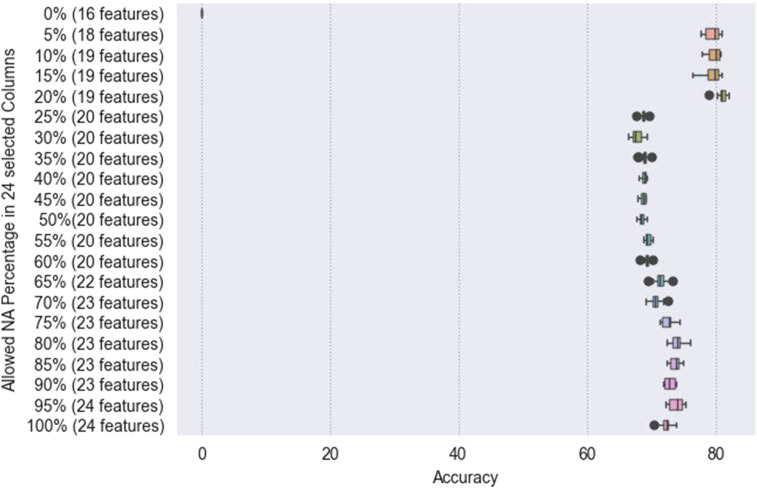
Comparison of the accuracy results for different percentage configurations (indicated number of (reduced) features due to incompleteness.

Incomplete datasets, for example, data generated through integration of various sources, exhibit missing values (NA). The model allows for elimination of missing values, based on the pandas library, by configuring the NA percentage in the config file. This function is highlighted in Figure 2. A combined dataset of several different studies combining 2967 samples has been used as an example holding incomplete features. Two age columns that have been named differently (Age, Age at First Diagnosis) had been combined to have one more complete numerical feature. Then 24 features were specified in the config and the percentage of NAs has been successively changed between intervals of 0%, 5%, 25%, 50%, 75%, and 100%. Accuracy was high at 100% and decreased with decreasing percentage of allowed NAs. At 5%, another peak of the highest accuracy could be observed, while 0% of NAs did not result in any data synthesis due to lacking numerical values from input data. In general, a low NA percentage also decreases the number of features if they are incomplete. In the example given the number of features decreased from 25 to 18 when going down from 100 to 5% NAs, and no output in case of 0%.

Model accuracy for synthetic data similarity can be influenced by the individual selection of features, to be set in the config file. On the one hand, accuracy is affected by the count of features, on the other hand, on the quality and completeness of selected features as can be observed in the previous example.

### Qualitative comparison of synthetic data

Next to quantitative results based on accuracy, the output can also be compared to observations reported in the literature. At least, synthetic data should reflect sample input data regarding the context between specific features.

**Figure 3.**
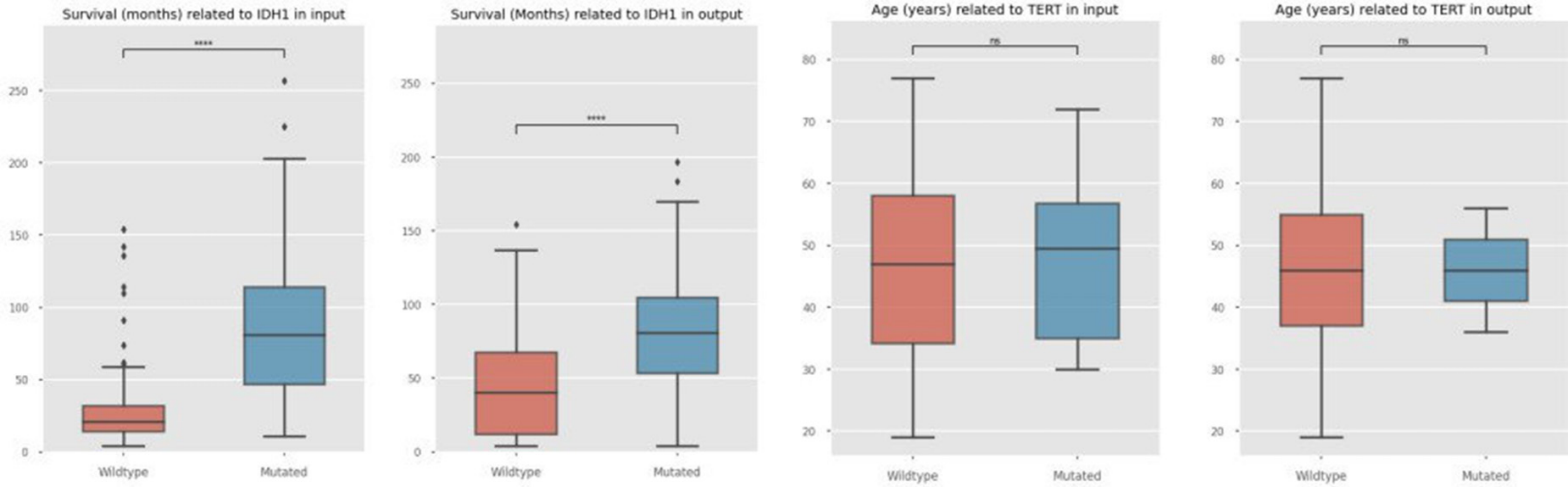
Comparison of selected samples from Diffuse Glioma (the GLASS Consortium, Nature 2019: difg_glass_2019) as input data (a) and synthetic output data (b) regarding WT and mutated IDH1 impact on survival, and sample input data (c) and synthetic output data (d) regarding WT and TERT aberrations in relation to age. WT: wildtype; TERT: telomerase reverse transcriptase.

**Figure 4.**
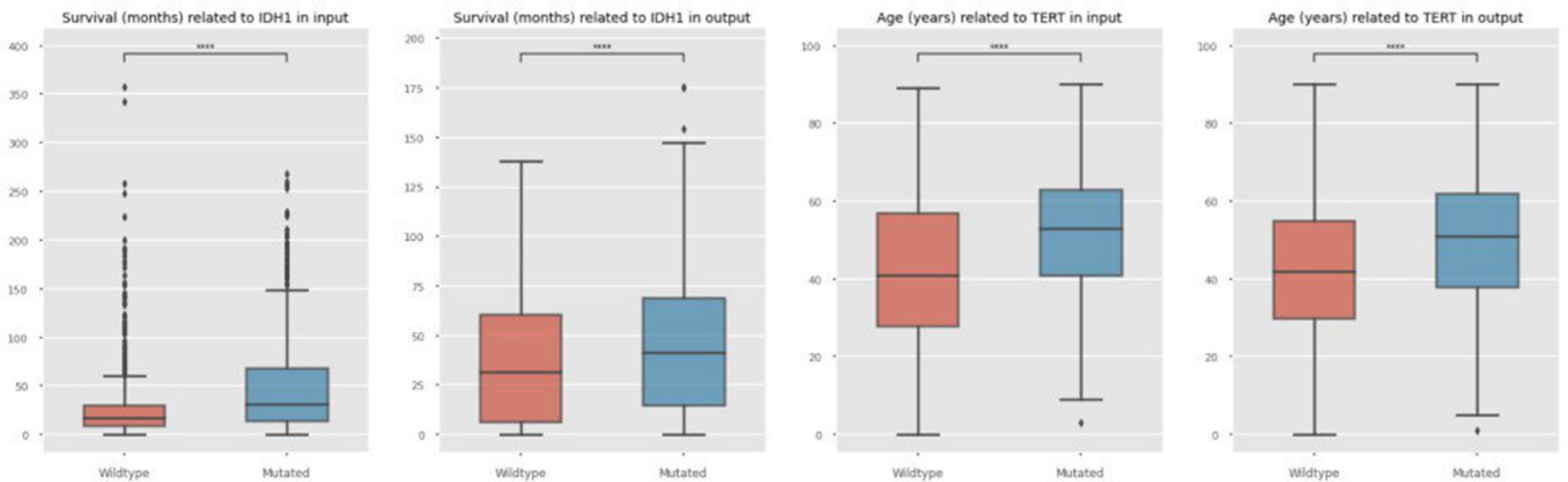
Comparison of selected samples from a combined dataset (brain_cptac_2020, difg_glass_2019, gbm_tcga, glioma_msk_2018, glioma_mskcc_2019, lgg_tcga, lgg_ucsf_2014) input data (a) and synthetic output data (b) regarding WT and mutated IDH1 impact on survival in combined datasets. Comparison of sample input data (c) and synthetic output data (d) regarding WT and TERT aberrations in relation to Age. WT: wildtype; TERT: telomerase reverse transcriptase.

In cases of diffuse glioma, it has been reported that mutations in the IDH genes are favourable prognostic markers.^89,90^ This means that samples with IDH1 mutations should show a higher number for survival in months, which can be observed in the sample as well as synthetic data shown in Figure 3 for one exemplary complete dataset as well as the combined dataset from multiple sources in Figure 4.

Another exemplary correlation that has been described specifies TERT gene mutations to be associated with older ages.^91-93^ In this case, no significant difference is observed in the case of using the complete dataset as input, in sample nor synthetic data, as shown in Figure 3. This result is also due to the low frequency of occurring TERT mutations among samples of the corresponding dataset by the glass consortium,^78^ including 14 out of 444 samples only, while for IDH1 the mutation frequency includes 174 out of the 444 samples. The combined data of multiple datasets includes studies with a higher mutation frequency of TERT resulting in significant differences in input and output data, shown in Figure 4.

### UX and feasibility for data sharing

We studied feasibility by conducting a TA test and collected answers from a SUS questionnaire in Table 1. The average SUS is 78 which relates to a good usability score. Our approach on https://github.com/dude2033/data_synthesizer presents an easy-to-use and adaptable Jupyter notebook, including a config file in JSON format with an example of a data input. All the files have been shared via container format, while test participants have been familiar with either CyVerse Austria or Jupyter. All participants were introduced to the task of generating data via the script and its main advantages or possibilities. All participants had a life science background. Tasks executed and tested included scenarios of simply running the sample scripts as well as applying them to their own tabular data in various formats (tsv/csv).

**Table 1.** SUS scores: Answers per participant (1 = strongly disagree, 5 = strongly agree).

I think that I would like to use this system frequently.	3	4	3	5	4
I found the system unnecessarily complex.	2	2	2	2	1
I thought the system was easy to use.	4	4	4	4	5
I think that I would need the support of a person to be able to use this system.	1	3	2	3	1
I found the various functions in this system were well integrated.	4	4	4	5	5
I thought there was too much inconsistency in this system.	5	1	1	2	1
I would imagine that most people would learn to use this system very quickly.	2	3	3	4	4
I found the system very cumbersome to use.	1	1	2	1	1
I felt very confident using the system.	4	4	4	4	4
I needed to learn a lot of things before I could get going with this system.	1	2	2	1	1
SUS Score	67.5	75	72.5	82.5	92.5

SUS: system utility score.

Key points noted through the qualitative tests include the necessity for comprehensive guidelines, either as accompanying documentation files or personal guide assisting new users in running the model the first time. A basic understanding of the platform is necessary to navigate through the system and run a shared analysis without such support.

## Discussion

The presented approach to generate synthetic data from various input datasets is intended to function as an exemplary use case for sharing controlled data as well as for data augmentation. The framework embeds a user-centric approach to improve the likelihood of data sharing.

This study presents a qualitative glance at the suitability of working with an accessible online platform such as CyVerse with Jupyter notebooks to support non-experts in generating synthetic data. It does not provide a comprehensive guide on how to generate synthetic data, but rather functions as motivation and description of one of the multiple possible ways to foster open science in biomedical data sharing. The ultimate goal is to support scientists in exchanging data in order to improve models for biomedical research and other domains that have to deal with privacy issues of sample data.

The utility of synthetic data generators is still under discussion and depends on data preprocessing steps on the one hand and data generation as well as usage settings on the other hand.^81^ The framework introduced herein allows to interpolate incomplete datasets in order to support data augmentation and preprocessing. Still, the synthetic data can only meet the criteria set by the sample data that has been used as input. The setting of NA percentages in the config file defines a minimum of filled data entries in the input data set to interpolate and fill incomplete data entries. Interpolation of data based on a low percentage of given input data entries will generate an accurate output to a lesser degree than complete data input. The highest accuracy was reached during the various runs presented in Figure 2.

Another consideration is based on a suitable total number of samples and a related equal size of categories therein. Also, in the case of complete data entries, there is still the possibility of some categories being underrepresented. An imbalance can lead to a problematic dataset ratio.^35^ In the exemplary single dataset used for the qualitative comparison of features it has been observed that TERT mutations are underrepresented compared to wildtype TERT. While connections between specific features have been observed as for the example of IDH1, others could not be replicated. This is reflected by the lack of significance in age and TERT correlation in both sample input and synthetic output, as shown in Figure 3.

The synthetic data vault framework has recently published a new major release integrating application programming interfaces for (semi)automatic metadata and feature extraction.^95,96^ The importance of simple and intuitive user interfaces has always been underestimated, however, are ultimately crucial for end-user acceptance, especially for future human–AI interfaces.^97^

We concentrated on the use of a separate config file to handle individual input data. The synthetic data vault’s newly promoted version^96^ presents a bunch of demos of how to generate synthetic data out of single- and multi-table as well as sequential data via Google Colab links. Ease of use also has its assets and drawbacks; for our use case, the new version’s demos do not provide a solution to support our configuration for NA Percentage and feature selection.

Additionally, the new release integrates visualization support to evaluate generated data. The underlying model-agnostic Python library SDmetrics, a part of the SDV project, allows to compare synthetic data against real data using a variety of metrics. Thereby, the SDMetrics library counts fairness, accuracy and explainability as top values based on a large open-source community including AI experts and intuitive and clear communication of underlying metrics.^98^ Its updated version allows to generate visual reports in order to increase model transparency and trust in generated data since ethical concerns have been implicated with AI systems demanding trustworthiness in regard to legislation, ethics, and robustness. One could also argue that synthetic data will always have the character of artificiality. The above-alluded issue of trustworthy AI can be circumvented by the use of synthetic data in the first place. It could even call for incentivizing the generation of high-quality synthetic data with due consideration of the disclosure of produced data and its generating processes accompanied by its proper indication and documentation.^99,100^ With respect to trustworthy AI, our general approach is based on the transparency of individual computing steps. The original intention was to answer the question of whether inexperienced users may be interested and aided by applying the tool. Additionally, the tool can also be used by experienced users to be able to easily inspect several steps of computations and understand the mechanics of the system.

As a prerequisite for the TA test, participants had to either be already familiar with or be guided through the container running process within the CyVerse system before starting with the actual TA test. Docker containers on the CyVerse platform can be started via the applications section or be rerun from the analyses section. This container was shared as a running Jupyter Notebook application with the corresponding workspace containing the readme file in the first tab and the script in the next tab. On the left hand, the folder content was detailed with the input file named combined.csv in addition. As soon as the output was written, a new file was presented in the same section.

The aim of this feasibility study was to examine if both the script could be executed (with given samples), and secondly, if the config file could be changed in order to apply its own dataset for generating synthetic data.

Proceeding with the TA test plan, participants guessed correctly, that introductory notes can be found within the readme file within the root of the container. However, the participants indicated that provided information could be improved. We therefore added further information regarding sample size and the handling of missing values (NAs) in Python.

All participants knew the concept of Jupyter notebooks and, therefore, also succeeded in running the script. However, some questions have been asked during the KScomplement script’s computational runtime. Therefore, we added further print lines with assistive information such as progress information, to better monitor what is going on. The target group of scientists with no IT experience could be better served by presenting a graphical user interface throughout the complete data generation process. Virtual lab books hold the advantage of directly presenting visualizations which are for now shown only as prints of process updates. The script’s passages have still to be run manually, while each Jupyter cell’s print output supports script transparency. The goal of feasibility testing was to learn from qualitative feedback. Therefore, the TA method was applied. SUS results are only a minor addition to post-test questionnaire and are not fully representative due to the variability of the target population, however, suitable for an early phase study. Eventually, all participants agreed on the system to be easy to use. The system could also support researchers in understanding their data when it comes to incompleteness and their possibilities to provide former sensitive material to be reusable and repurposable by a wider research community.

## Conclusions

This framework is based on multivariate modeling for data generation and an integrated function for interpolating incomplete data sources. It is provided as a computational notebook workspace inside a container run in an institutional cyberinfrastructure platform in order to better serve scientists lacking a certain IT background. The UX study shows that test participants both approve as well as prefer web-based mechanisms such as CyVerse and Jupyter over desktop platforms, since they are not limited to a specific local infrastructure setting. Providing (qualitative) data from patients is vital to moving forward in cancer research, which is likewise true for other medical domains as well as distinct disciplines. The given system can be expanded from medical data to any other domain where synthetic data is needed. This work can also be used to improve the quality of data, for example, the usage of synthetic data to mitigate bias in training datasets (e.g. increasing amount of data for underrepresented groups). The framework is presented as a computational notebook workspace inside a shareable container avoiding environment and version issues, still, a graphical user interface guiding users through the complete data generation process could be implemented in future to overcome the limitation of the script-based system. This framework presents an integrated configuration function for tabular data only. Further examples could be developed involving multiple formats as input, and, moreover, support users to provide data openly. Future studies could also assess the different user needs due to various domain specifics, levels of prior knowledge, incompleteness, or sensitivity issues.

## Supplemental Material

sj-pdf-1-dhj-10.1177_20552076241271769 - Supplemental material for Unlocking biomedical data sharing: A structured approach with digital twins and artificial intelligence (AI) for open health sciences Supplemental material, sj-pdf-1-dhj-10.1177_20552076241271769 for Unlocking biomedical data sharing: A structured approach with digital twins and artificial intelligence (AI) for open health sciences by Claire Jean-Quartier, Sarah Stryeck, Alexander Thien, Burim Vrella, Jeremias Kleinschuster, Emil Spreitzer, Mojib Wali, Heimo Mueller, Andreas Holzinger and Fleur Jeanquartier in DIGITAL HEALTH
