# Supplementary material for: Unlocking biomedical data sharing: A structured approach with digital twins and artificial intelligence (AI) for open health sciences
Source: Digit Health. 2024 Sep 5;10:20552076241271769. doi: 10.1177/20552076241271769 (PMC11394355; doi:10.1177/20552076241271769)
Supplement: sj-pdf-1-dhj-10.1177_20552076241271769 - Supplemental material for Unlocking biomedical data sharing: A structured approach with digital twins and artificial intelligence (AI) for open health sciences [file sj-pdf-1-dhj-10.1177_20552076241271769.pdf]

Test procedure

The Thinking Aloud test comprises tasks performed by selected test users who are observed during the test while performing a set of the following tasks verbally expressing their thoughts out loud. In other words – users are asked to say everything they **see, think, do, and feel** at any given moment.

Tests are conducted remotely via the video conference software Webex. Each meeting with three people: A Moderator – the biomedical expert, the observer – a user experience expert, and the test user.

Prerequisite

Test user and moderator create a Cyverse Account (if not already registered), login to the system, share container and get a quick introduction to CyVerse (if not already acquainted).

Questions & Tasks

1.) As starting questions, let test participants introduce themselves: Please shortly describe which background knowledge do you have on data science and in the biomedical domain. Do you work with large amounts of data? Are you familiar with python?

2.) Task 1: Please log in to Cyverse, start the container, and read the instructions in the readme file. Tell us what you think while reading and which questions come into your mind. Do you understand what is possible with this tool?

3.) Task 2: Please open the config file. Tell us for each configuration, what you believe it is for!

4.) Task 3: Now, please try to change some of the configurations by first having a glance at the data:
    a. Either have a look at the file named combined.tsv.
    b. Or you have a reduced textual data spreadsheet of yours at hand, that you would like to use.
    ⇨ Now try to change the input parameters according to the column names and value types corresponding the chose spreadsheet. Tell us your thoughts on the function of the configuration!

5.) Task 4: Finally, open the notebook as stated in the instructions and run it. Again, tell us what you believe you are doing before and/or during each interaction with the notebook. Please also read all the comments that can be found in the notebook and tell us your thoughts!

Post Questionnaire

SUS-Test (Standard)

https://doi.org/10.1080/10447318.2018.1455307 - Figure 1.
